# Beyond physical distance: how workplace loneliness links telecommuting to cyberloafing

**DOI:** 10.3389/fpsyg.2026.1978751

**Published:** 2026-10-15

**Authors:** Usman Ghani, Yongnian Su, Yangyang Shi, Yufei Zhou, Jin Cheng

**Affiliations:** 1 Iqra University Business School, Iqra University, Karachi, Pakistan; 2 School of Management, Xiamen University, Xiamen, China; 3 School of Journalism and Communication, Xiamen University, Xiamen, China; 4 Panyapiwat Institute of Management, Pak Kret, Nonthaburi, Thailand

**Keywords:** conservation of resources theory, cyberloafing, leader-member exchange, task interdependence, telecommuting, workplace loneliness

## Abstract

Telecommuting offers flexibility, but it may also be accompanied by fewer opportunities for everyday social connection in the workplace. Drawing on Conservation of Resources theory, this study examines how such social disconnection is associated with cyberloafing and why this process varies across employees. Using two-wave survey data from 350 employees in China with recent telecommuting experience, we found a significant indirect association between telecommuting and cyberloafing through workplace loneliness. This indirect association was weaker when employees reported higher-quality leader–member exchange or greater task interdependence. These two conditions reflect different forms of workplace resources: leader–member exchange provides relational support, whereas task interdependence sustains interaction through the organization of work itself. These findings shift attention away from whether telecommuting is inherently beneficial or harmful and toward the social resource conditions that shape employees’ responses to working apart. They also offer a more nuanced view of cyberloafing as a possible short-term withdrawal-based digital response that is consistent with coping interpretations under conditions of diminished workplace connection.

## Introduction

Telecommuting has become an established feature of contemporary work, allowing employees to perform their jobs away from conventional workplaces through digital technologies (Allen et al., 2015; Wang et al., 2021). Yet its consequences are far from uniform. Remote work can offer flexibility and autonomy while reducing opportunities for spontaneous interaction, informal communication, and everyday social contact (Gajendran and Harrison, 2007; Ipsen et al., 2021; Walz et al., 2024). Research on remote and virtual work has similarly highlighted risks of professional isolation and weakened informal workplace ties when employees are physically dispersed (Cooper and Kurland, 2002; Bartel et al., 2012; Raghuram et al., 2019). Thus, physical distance alone may not determine whether telecommuting is beneficial or detrimental; what matters is whether employees remain socially connected and supported while working apart (Cheng and Zhang, 2022; McCarthy et al., 2026). This raises a central question: how may employees’ access to valued workplace social resources differ under reduced physical co-presence, and how might employees respond when such resources become less accessible?

Cyberloafing may represent one such response. It refers to employees’ use of work time or digital resources for personal, non-work-related online activities (Lim and Chen, 2012; Askew et al., 2014; Blanchard and Henle, 2008). Although traditionally treated as counterproductive behavior, cyberloafing may also arise in response to workplace stressors, boredom, or resource depletion (Andel et al., 2019; Pindek et al., 2018; Zhang et al., 2022). Rather than viewing it solely as deliberate misconduct, we consider cyberloafing a possible withdrawal-based digital response that is consistent with coping interpretations, through which employees may temporarily disengage from work or seek relief from negative work experiences. Importantly, this interpretation does not assume that cyberloafing is necessarily an intentional coping strategy; rather, it recognizes that non-work digital activity may emerge as one form of temporary disengagement under conditions of workplace strain.

Workplace loneliness provides a potential psychological mechanism linking telecommuting with such digital withdrawal. It reflects employees’ perceived insufficiency of meaningful social relationships and workplace connection (Ozcelik and Barsade, 2018; Wright and Silard, 2021) and has increasingly been recognized as consequential for employees’ psychological and work experiences (Tian et al., 2023; McCarthy et al., 2026). Telecommuting is not inevitably accompanied by loneliness, but reduced face-to-face contact may limit informal interaction and relationship maintenance that ordinarily provide valued social resources (Cheng et al., 2023; Walz et al., 2024). From a Conservation of Resources (COR) perspective, employees seek to preserve such resources (Hobfoll, 1989; Hobfoll et al., 2018; Halbesleben et al., 2014). Accordingly, workplace loneliness is not treated here merely as a negative affective reaction to working remotely, but as a subjective psychological experience of insufficient access to valued workplace social resources, including everyday interaction, relational contact, and a sense of belonging. When access to these resources weakens, workplace loneliness may emerge as a psychological manifestation of social-resource insufficiency and may be associated with greater temporary digital withdrawal (Andel et al., 2019; Zhang et al., 2022).

Whether this process unfolds, however, may depend on the resources available within employees’ work environment. This study focuses on leader–member exchange (LMX) and task interdependence as two distinct resource conditions. High-quality LMX provides relational resources through trust, feedback, and socio-emotional support (Graen and Uhl-Bien, 1995; Dulebohn et al., 2012), which may become especially salient when everyday face-to-face access to supervisors is reduced (Golden and Veiga, 2008; Varma et al., 2022). Task interdependence, by contrast, functions as a structural or work-design resource by embedding communication, coordination, and mutual reliance into the work process (Campion et al., 1993; Wageman, 1995). Interdependent work therefore creates recurring opportunities for interaction even when employees work apart (Van der Vegt and Van de Vliert, 2005; Cheng et al., 2023). These two conditions represent different ways in which workplace social resources may remain accessible despite physical separation: LMX preserves connection through the quality of the supervisor–employee relationship, whereas task interdependence preserves interaction through the organization of work itself.

Against this background, the present study examines whether telecommuting is associated with cyberloafing through workplace loneliness and whether this indirect relationship varies according to LMX and task interdependence. Drawing on COR theory, we examine these relationships within a social-resource framework while distinguishing LMX as a relationship-based resource and task interdependence as a work-design-based resource. Recent research has shown that flexible work arrangements may heighten workplace loneliness and thereby shape employees’ interpersonal behavior, with task interdependence serving as an important boundary condition (Cheng et al., 2023). Relatedly, the present study develops a resource-based account focused specifically on telecommuting and reduced physical co-presence, asking how diminished access to workplace social resources may become reflected in digital withdrawal and how different forms of workplace resources may interrupt this process. The proposed research model is presented in Figure 1.

**Figure 1:**
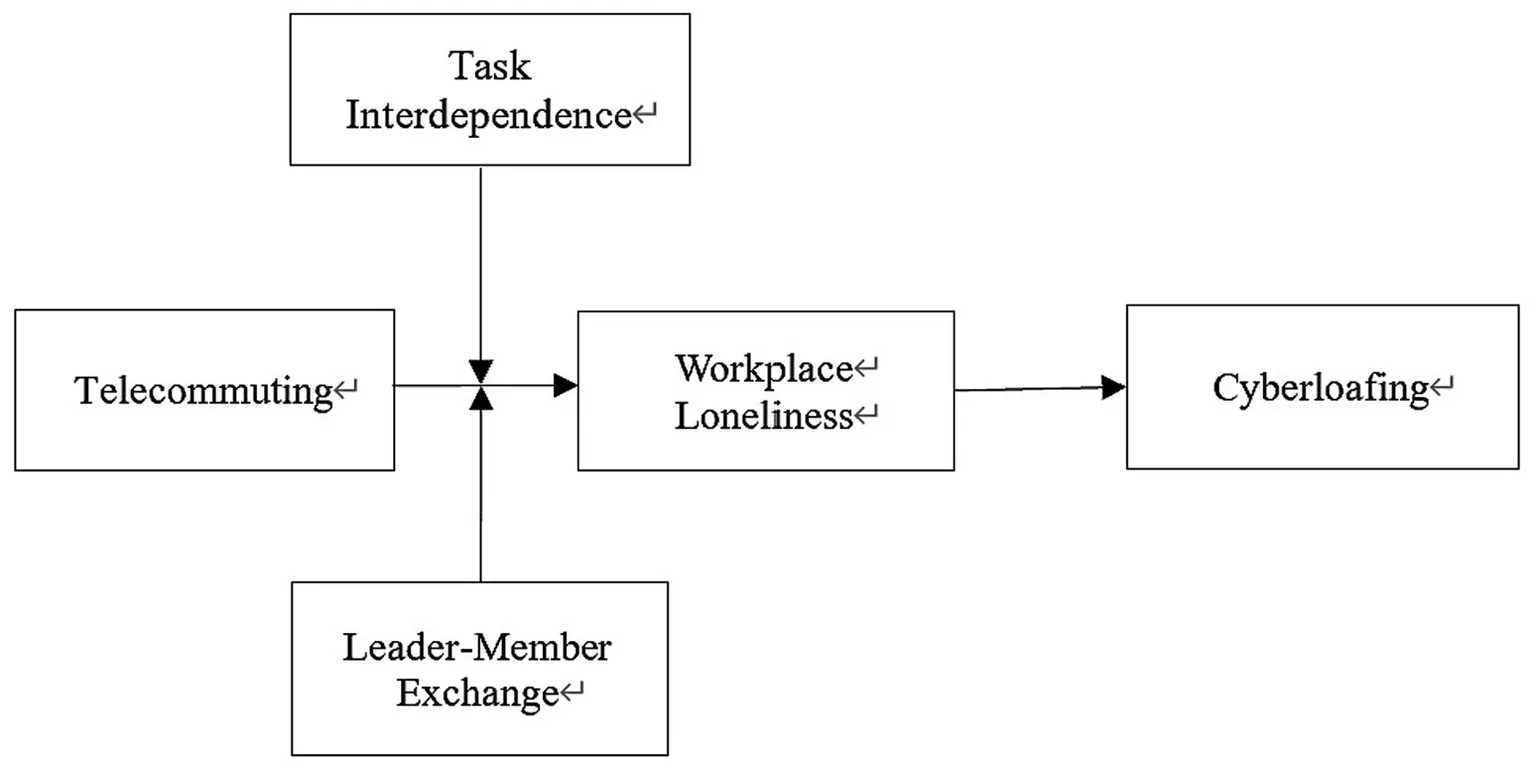
Proposed research model.

In doing so, this study contributes to the telecommuting literature in three ways. First, it develops a resource-based explanation of telecommuting by focusing on the availability and preservation of workplace social resources under reduced physical co-presence. In this account, workplace loneliness represents the subjective experience through which diminished access to social resources becomes psychologically consequential. Second, the study extends the behavioral consequences of workplace loneliness beyond reduced interpersonal engagement. Rather than examining only what lonely employees may become less willing to contribute to coworkers, it considers whether social-resource strain is also associated with temporary disengagement through non-work digital activity. Cyberloafing is therefore interpreted cautiously as a withdrawal-based digital response consistent with coping interpretations rather than as direct evidence of intentional coping. Third, the study distinguishes two theoretically distinct resource-buffering pathways. LMX represents a relationship-based resource, whereas task interdependence represents a work-design-based resource, allowing us to examine whether workplace connection can be preserved through interpersonal relationships, the organization of work, or both.

## Hypotheses development

### Conservation of resources theory

COR theory provides the overarching framework for the proposed model because the central question of this study concerns the availability, loss, and preservation of workplace social resources under telecommuting. COR theory holds that individuals strive to obtain, retain, and protect valued resources, and that actual or threatened resource loss can generate strain and motivate efforts to conserve remaining resources (Hobfoll, 1989; Halbesleben et al., 2014; Hobfoll et al., 2018). COR theory further emphasizes that individuals invest existing resources to protect against resource loss and maintain other valued resources, while resource losses may become more consequential when compensatory resources are limited (Hobfoll et al., 2018). In the present study, LMX and task interdependence are therefore viewed as contextual resources that may help employees preserve access to workplace social resources when physical co-presence is reduced. Under telecommuting, reduced physical co-presence may constrain employees’ access to social resources embedded in everyday workplace interaction, whereas relational and work-design conditions may help preserve such resources when employees work apart.

Other theoretical perspectives can illuminate particular aspects of these relationships. Affective events theory emphasizes how workplace events evoke affective reactions (Weiss and Cropanzano, 1996), self-determination theory highlights the consequences of satisfying or frustrating basic psychological needs, including relatedness (Deci and Ryan, 2000), and social exchange theory focuses on reciprocal social relationships and exchange obligations (Blau, 1964). COR theory is particularly suited to the present model because it provides an integrative account of how workplace social resources may become less accessible under telecommuting and how different resource conditions may help preserve access to these resources.

### Telecommuting and workplace loneliness

Telecommuting represents a specific form of flexible work centered on where work is performed. Whereas the broader concept of flexible work arrangements may encompass both temporal and locational flexibility, the present study focuses specifically on the extent to which employees perform their work away from the conventional organizational workplace (Bailey and Kurland, 2002; Allen et al., 2015; Wang et al., 2021).

Workplace loneliness reflects employees’ perceived insufficiency of meaningful social connection and interpersonal relationships at work (Ozcelik and Barsade, 2018; Wright and Silard, 2021; McCarthy et al., 2026). Consistent with classic loneliness research, loneliness arises not simply from objective isolation but from a discrepancy between desired and available social relationships (Weiss, 1973). Weiss (1973) distinguished social loneliness, arising from insufficient social integration, from emotional loneliness, arising from the absence of close and meaningful relational bonds. Although this distinction provides useful conceptual background, the present study treats workplace loneliness as an overall subjective experience of insufficient workplace connection. This distinction is especially relevant to telecommuting, where physical separation does not necessarily result in loneliness unless employees perceive their workplace relationships and social connection as insufficient.

According to COR theory, employees seek to acquire and maintain valued resources, including social resources embedded in workplace relationships (Hobfoll, 1989; Hobfoll et al., 2018; Halbesleben et al., 2014). When telecommuting reduces opportunities for informal interaction, relational contact, and social support, employees may perceive valued workplace social resources as less accessible. Workplace loneliness can therefore be understood not simply as an affective reaction to working remotely, but as a subjective psychological experience associated with insufficient access to desired workplace social connection. Prior research has similarly emphasized that the social consequences of remote work depend on the availability of interaction, support, and connection rather than on physical separation alone (Cheng et al., 2023; Fostervold et al., 2024; O’Hare et al., 2024). From this resource-based perspective, telecommuting becomes consequential when reduced physical co-presence is accompanied by diminished access to valued social resources. Thus, telecommuting is expected to be positively associated with workplace loneliness.

Based on these arguments, we propose the following hypothesis:

*Hypothesis 1*: Telecommuting is positively associated with workplace loneliness.

### Workplace loneliness and cyberloafing

Workplace loneliness reflects a perceived lack of meaningful social connection at work and may be associated with psychological strain when employees’ relational needs remain unmet (Baumeister and Leary, 1995; Wright and Silard, 2021; Tian et al., 2023). From a COR perspective, such strain may signal insufficient access to valued social and emotional resources, prompting employees to conserve remaining resources or temporarily withdraw from demanding work activities (Hobfoll et al., 2018; Halbesleben et al., 2014). In this context, cyberloafing may represent a short-term withdrawal-based digital response, through which employees temporarily disengage from work demands (Henle and Blanchard, 2008; Mercado et al., 2017).

Prior research has linked cyberloafing to workplace stressors, aggression, boredom, and emotional exhaustion (Andel et al., 2019; Pindek et al., 2018; Zhang et al., 2022). These findings suggest that cyberloafing may occur not only as intentional counterproductive behavior but also in contexts of psychological strain and temporary disengagement. Workplace loneliness may reflect such a strain context by signaling insufficient social connection at work. Accordingly, employees who experience greater workplace loneliness may be more likely to engage in cyberloafing as a form of short-term digital withdrawal.

Based on these arguments, we propose the following hypothesis:

*Hypothesis 2*: Workplace loneliness is positively associated with cyberloafing.

### The mediating role of workplace loneliness

Taken together, the preceding arguments suggest that workplace loneliness may explain how telecommuting is associated with cyberloafing. Reduced access to everyday social interaction and relational resources under telecommuting may be associated with stronger feelings of insufficient workplace connection, which may in turn be accompanied by greater temporary disengagement through non-work digital activities (Golden et al., 2008; Sardeshmukh et al., 2012; Tian et al., 2023). From a COR perspective, this process reflects a resource-based pathway in which reduced access to workplace social resources is associated with workplace loneliness, which may subsequently be accompanied by greater digital withdrawal (Hobfoll, 1989; Hobfoll et al., 2018). Workplace loneliness therefore represents a psychological pathway linking diminished workplace connection with cyberloafing. Based on these arguments, we propose the following hypothesis:

*Hypothesis 3*: Workplace loneliness mediates the positive association between telecommuting and cyberloafing.

### The moderating role of leader–member exchange

LMX reflects the quality of the relationship between employees and their supervisors, with high-quality exchanges characterized by trust, support, mutual respect, and access to valued resources (Graen and Uhl-Bien, 1995; Dulebohn et al., 2012). In telecommuting contexts, where opportunities for informal face-to-face interaction with supervisors are reduced, such relationships may become especially important for maintaining employees’ sense of workplace connection (Golden and Veiga, 2008; Varma et al., 2022). From a COR perspective, high-quality LMX can therefore be understood as a relationship-based resource, providing employees with attention, feedback, and socio-emotional support when everyday in-person contact is limited (Eisenberger et al., 1986; Rhoades and Eisenberger, 2002). Unlike work-design resources that create interaction through task structure, LMX preserves social connection through the quality of the supervisor–employee relationship itself. These resources may help employees sustain a sense of connection despite reduced physical co-presence, which may be associated with a weaker positive relationship between telecommuting and workplace loneliness.

Based on these arguments, we propose the following hypothesis:

*Hypothesis 4*: LMX negatively moderates the positive association between telecommuting and workplace loneliness, such that the association is weaker when LMX is high.

Because workplace loneliness constitutes the mediating mechanism proposed in H3, this buffering role should also extend to the indirect association between telecommuting and cyberloafing. When LMX is high, stronger relationship-based resources may weaken the first stage of this indirect process; when LMX is low, the indirect association through workplace loneliness should be stronger.

Therefore, we propose:

*Hypothesis 5*: LMX moderates the indirect association between telecommuting and cyberloafing through workplace loneliness, such that the indirect association is weaker when LMX is high and stronger when LMX is low.

### The moderating role of task interdependence

Task interdependence reflects the extent to which employees rely on one another for information, coordination, and task completion (Campion et al., 1993; Wageman, 1995). In telecommuting contexts, high task interdependence can function as a structural or work-design-based resource because it embeds communication and mutual reliance into the work process itself (Van der Vegt and Van de Vliert, 2005; Triana et al., 2025). Even when employees are physically dispersed, interdependent tasks create recurring opportunities for interaction and coordination, helping sustain workplace connection despite reduced physical co-presence (Chiaburu and Harrison, 2008; Sias and Cahill, 1998). This work-design pathway differs from LMX, which preserves connection through the quality of the supervisor–employee relationship. From a COR perspective, these work-embedded interactions may help preserve access to valued social resources and may therefore be associated with a weaker relationship between telecommuting and workplace loneliness (Hobfoll et al., 2018).

Prior research has similarly shown that task interdependence can buffer loneliness-related consequences under flexible work arrangements (Cheng et al., 2023). Within the present COR-based framework, task interdependence is treated as a distinct work-design resource alongside the relationship-based resources provided by LMX.

Based on these arguments, we propose the following hypothesis:

*Hypothesis 6*: Task interdependence negatively moderates the positive association between telecommuting and workplace loneliness, such that the association is weaker when task interdependence is high.

As with LMX, this buffering role should extend to the indirect association proposed in H3. When task interdependence is high, work itself provides recurring opportunities for communication and coordination, weakening the first stage of the indirect process; when task interdependence is low, the indirect association through workplace loneliness should be stronger.

Therefore, we propose:

*Hypothesis 7*: Task interdependence moderates the indirect association between telecommuting and cyberloafing through workplace loneliness, such that the indirect association is weaker when task interdependence is high and stronger when task interdependence is low.

## Method

### Sample and procedure

The target population was employees in China with recent telecommuting experience. Participants were recruited through a major online survey platform using convenience sampling. Participation was voluntary, and respondents self-selected into the study. To ensure sample relevance, respondents were required to confirm that they: (1) were currently employed, (2) had a direct supervisor, (3) primarily used a computer and the internet for work, and (4) had engaged in telecommuting within the previous month. Participants provided informed consent and were assured that their responses would remain confidential and anonymous.

The study employed a two-wave time-lagged survey design with a two-week interval to introduce temporal separation and capture relatively proximal workplace experiences. At Time 1 (T1), participants reported telecommuting intensity, leader–member exchange, task interdependence, and demographic information. Two weeks later, the Time 2 (T2) survey assessed workplace loneliness and cyberloafing. Responses were matched across waves using anonymous participant identifiers.

At T1, 452 responses were collected through the online survey platform. Responses were screened for both eligibility and data quality. We excluded 18 respondents because they did not meet the predefined eligibility criteria. An additional 32 responses were removed because of data-quality concerns, including abnormally short completion times, straight-lining response patterns, and otherwise invalid submissions. Following this screening, 402 valid T1 respondents remained and were invited to participate in the second-wave survey.

Two weeks later, 367 respondents completed the T2 survey, corresponding to a follow-up rate of 91.3% and a wave attrition rate of 8.7%. During final screening, 9 T2 responses were excluded because they failed the data-quality criteria, and 8 responses could not be successfully matched with valid T1 records. Seventeen additional cases were therefore removed, resulting in a final analytic sample of 350 matched respondents.

To assess potential attrition bias, we compared Wave 2 completers with Wave 1 attriters on demographic characteristics and Wave 1 focal variables. Chi-square tests were used for categorical variables, and independent-samples t-tests were used for continuous variables. No statistically significant differences were identified between the two groups across the examined variables.

The demographic characteristics of the final sample are presented in Table 1. The sample was relatively balanced by gender (50.9% male), with most respondents aged between 25 and 45 years. Most participants held a bachelor’s degree (75.1%), and the majority reported between one and ten years of work experience. Detailed demographic information is provided in Table 1.

**Table 1:** Sample characteristics (*N* = 350).

Variable	Category	Frequency	Percentage
Gender	Male	178	50.9%
	Female	172	49.1%
Age	Younger than 25 years	40	11.4%
	25–35 years	166	47.4%
	35–45 years	113	32.3%
	45–55 years	23	6.6%
	Older than 55 years	8	2.3%
Education	Associate degree or below	53	15.1%
	Bachelor’s degree	263	75.1%
	Master’s degree or above	34	9.7%
Work experience	One year or less	11	3.1%
	One to five years	164	46.9%
	Five to ten years	102	29.1%
	Ten to fifteen years	59	16.9%
	More than fifteen years	14	4.0%

### Measures

As the survey was administered to Chinese employees, the measures were translated following Brislin’s (1980) back-translation procedure. The original English measures were translated into Mandarin Chinese by two bilingual experts and independently back-translated into English by another two bilingual experts. Discrepancies were resolved to ensure conceptual equivalence. Except for telecommuting, all multi-item constructs were assessed using five-point Likert-type scales, with higher scores indicating higher levels of the corresponding construct.

*Telecommuting.* Telecommuting intensity was measured as a single-item observed variable following Golden and Veiga (2005). Participants were asked, “How many hours did you work remotely on average per week during the past month?” This measure captures the weekly duration and extent of employees’ recent exposure to telecommuting. Because it represents a directly reported behavioral quantity rather than a multi-item latent construct, it was treated as an observed variable in subsequent analyses.

*Workplace loneliness.* Workplace loneliness was measured using a 12-item scale adapted from Wright (2005). The scale assessed perceived social disconnection, emotional distance, and lack of belonging at work. A sample item is, “I often feel alienated from my coworkers.”

*Cyberloafing.* Cyberloafing was measured using an 8-item scale developed by Lim (2002). The items assessed the frequency of non-work-related online activities during working hours while telecommuting. A sample item is, “While working remotely, I visited non-work-related websites, such as news, entertainment, or sports websites.”

*Leader–member exchange.* LMX was measured using the 7-item scale developed by Graen and Uhl-Bien (1995). A sample item is, “To what extent does your supervisor understand your work difficulties and needs?”

*Task interdependence.* Task interdependence was measured using a 3-item scale developed by Campion et al. (1993). A sample item is, “I could not have done my job without the information or materials provided by my coworkers.”

## Results

### Measurement model, reliability, and validity

Before testing the hypotheses, we assessed the measurement properties of the study constructs. Because telecommuting was measured using a single item, it was treated as an observed variable and excluded from the CFA, reliability, CR, and AVE analyses. The measurement model therefore included four multi-item latent constructs: workplace loneliness, cyberloafing, LMX, and task interdependence.

CFA was conducted using AMOS v22.0. As shown in Table 2, the hypothesized four-factor model demonstrated acceptable fit to the data (*χ*^2^ = 773.122, *df* = 399, *χ*^2^/*df* = 1.938, CFI = 0.945, TLI = 0.940, RMSEA = 0.052). The four-factor model also showed better fit than the alternative three-factor, two-factor, and one-factor models, supporting the distinctiveness of the four latent constructs.

**Table 2:** Confirmatory factor analysis model comparison.

Models	*χ* ^2^	*df*	*χ*^2^/*df*	CFI	TLI	RMSEA
4-factor model (Workplace loneliness, Cyberloafing, Leader–member exchange, and Task Interdependence)	773.122	399	1.938	0.945	0.940	0.052
3-factor model A (Workplace loneliness, Cyberloafing, Leader–member exchange + Task interdependence)	1,084.977	402	2.699	0.899	0.891	0.070
3-factor model B (Workplace loneliness + Cyberloafing, Leader–member exchange, and Task interdependence)	1,901.511	402	4.730	0.779	0.761	0.103
2-factor model (Workplace loneliness + Cyberloafing, Leader–member exchange + Task interdependence)	2,212.703	404	5.477	0.733	0.713	0.113
1-factor model (single-factor model)	3,457.460	405	8.537	0.550	0.516	0.147

Telecommuting was measured using a single item and was therefore excluded from the CFA. The plus sign (+) indicates constructs combined into a single factor.

Table 3 reports the standardized factor loadings, Cronbach’s alpha coefficients, CR, and AVE values. Factor loadings ranged from 0.601 to 0.886, Cronbach’s alpha values from 0.795 to 0.952, and CR values from 0.807 to 0.952. AVE values ranged from 0.544 to 0.625. All values met the recommended criteria, indicating satisfactory reliability and convergent validity (Fornell and Larcker, 1981; Hair et al., 2010). Discriminant validity was further assessed using the Fornell–Larcker criterion. As shown in Table 4, the square root of the AVE for each multi-item construct exceeded its correlations with the other constructs, providing further evidence of discriminant validity (Fornell and Larcker, 1981).

**Table 3:** Factor loadings, reliability, and convergent validity.

Variables	Items	Factor loadings	Cronbach’s alpha	CR	AVE
Workplace loneliness	WL1	0.741	0.952	0.952	0.625
	WL2	0.816			
	WL3	0.828			
	WL4	0.813			
	WL5	0.801			
	WL6	0.802			
	WL7	0.774			
	WL8	0.775			
	WL9	0.746			
	WL10	0.828			
	WL11	0.790			
	WL12	0.768			
Cyberloafing	CL1	0.754	0.902	0.904	0.544
	CL2	0.708			
	CL3	0.800			
	CL4	0.801			
	CL5	0.802			
	CL6	0.601			
	CL7	0.775			
	CL8	0.630			
Leader–member exchange	LMX1	0.708	0.912	0.916	0.610
	LMX2	0.815			
	LMX3	0.775			
	LMX4	0.761			
	LMX5	0.715			
	LMX6	0.828			
	LMX7	0.854			
Task interdependence	TI1	0.782	0.795	0.807	0.587
	TI2	0.886			
	TI3	0.604			

CR = composite reliability; AVE = average variance extracted. All reported factor loadings are standardized.

**Table 4:** Descriptive statistics and correlations.

Variable	Mean	SD	1	2	3	4	5	6	7	8	9
1. Gender	1.49	0.50	1								
2. Age	2.41	0.86	−0.108^*^	1							
3. Education	1.95	0.49	−0.111^*^	0.005	1						
4. Experience	2.72	0.92	−0.108^*^	0.751^**^	−0.015	1					
5. Telecommuting	22.26	12.19	−0.114^*^	0.072	−0.069	−0.013	1				
6. Workplace loneliness	2.12	0.87	−0.123^*^	−0.038	−0.037	−0.023	0.121^*^	0.**791**			
7. Leader–member exchange	3.71	0.76	−0.046	0.038	0.109^*^	0.102	−0.004	−0.361^**^	0.**781**		
8. Task interdependence	3.70	0.86	0.042	0.050	0.020	0.124^*^	0.009	−0.204^**^	0.255^**^	0.**766**	
9. Cyberloafing	2.33	0.82	−0.092	−0.077	−0.097	−0.074	0.182^**^	0.393^**^	−0.204^**^	−0.036	0.**738**

*N* = 350, ^*^p < 0.05, ^**^p < 0.01. Values in bold on the diagonal are the square roots of the average variance extracted (AVE) for the multi-item constructs. Telecommuting was measured using a single item; therefore, AVE was not applicable.

To reduce potential common method bias, the study employed a two-wave time-lagged design that temporally separated the measurement of antecedent and moderator variables from the mediator and outcome variables (Podsakoff et al., 2003). Harman’s single-factor test was also conducted as a preliminary diagnostic procedure. The first unrotated factor accounted for 33.70% of the total variance, which was below the commonly used benchmark of 50%, suggesting that no single factor dominated the covariance among the measures. In addition, a CFA model including a common method factor was estimated (Malhotra et al., 2006). The inclusion of the common method factor resulted in only marginal improvements in model fit: *χ*^2^ = 689.065, *df* = 369, *χ*^2^/*df* = 1.867, CFI = 0.946, TLI = 0.942, and RMSEA = 0.049. The limited improvement in model fit suggests that common method bias was unlikely to substantially bias the results.

### Descriptive statistics and correlations

Table 4 presents the means, standard deviations, and correlations among the study variables. Overall, the pattern of correlations among the focal variables was generally consistent with the expected directions.

### Mediation analysis

The mediating role of workplace loneliness was examined using PROCESS Macro Model 4 in SPSS 22 (Hayes, 2018). All focal variables were standardized before the PROCESS analyses, and standardized regression coefficients (*β*) are therefore reported. Gender, age, education, and work experience were included as control variables in PROCESS analyses. Indirect effects were estimated using 5,000 bootstrap samples with 95% confidence intervals.

As shown in Table 5, telecommuting was positively associated with workplace loneliness (*β* = 0.121, SE = 0.053, *t* = 2.271, *p* = 0.024), supporting H1. Workplace loneliness was also positively associated with cyberloafing (*β* = 0.376, SE = 0.049, *t* = 7.646, *p* < 0.001), supporting H2. The total effect of telecommuting on cyberloafing was positive and significant (*β* = 0.174, SE = 0.053, 95% CI [0.066, 0.276]). After workplace loneliness was included in the model, the direct effect remained significant (*β* = 0.129, SE = 0.050, 95% CI [0.033, 0.229]). The indirect effect of telecommuting on cyberloafing through workplace loneliness was 0.045 (Boot SE = 0.022, 95% BootCI [0.006, 0.095]). Because the bootstrap confidence interval excluded zero, H3 was supported. The significant direct and indirect effects indicate a partial mediation pattern. Although statistically significant, the magnitude of the indirect effect was relatively modest, suggesting that workplace loneliness provides a meaningful but limited mediating pathway between telecommuting and cyberloafing.

**Table 5:** Mediation analysis results.

Path	*β*	SE	*t*	*p*
Telecommuting → Workplace loneliness	0.121	0.053	2.271	0.024
Workplace loneliness → Cyberloafing	0.376	0.049	7.646	<0.001

Effect type	Effect	SE	LLCI	ULCI
Total effect (Telecommuting → Cyberloafing)	0.174	0.053	0.066	0.276
Direct effect (Telecommuting → Cyberloafing)	0.129	0.050	0.033	0.229

Effect type	Effect	Boot SE	BootLLCI	BootULCI
Indirect effect (Telecommuting → Workplace loneliness → Cyberloafing)	0.045	0.022	0.006	0.095

Bootstrap sample size = 5,000. LLCI = lower limit confidence interval; ULCI = upper limit confidence interval; BootLLCI = bootstrap lower limit confidence interval; BootULCI = bootstrap upper limit confidence interval.

As supplementary robustness checks, we re-estimated the mediation model without demographic controls and after excluding respondents reporting no more than 8 h of telecommuting per week. In both analyses, the indirect effect remained statistically significant and highly similar in magnitude to the main result, suggesting that the focal indirect association was not sensitive to the inclusion of control variables or to respondents with very limited telecommuting exposure.

### Moderation and moderated mediation analysis

The moderating effects of LMX and task interdependence on the association between telecommuting and workplace loneliness were examined using PROCESS Macro Model 1. The corresponding conditional indirect effects were further tested using PROCESS Macro Model 7, with 5,000 bootstrap samples and 95% confidence intervals.

As shown in Table 6, the interaction between telecommuting and LMX was significant (*β* = −0.098, SE = 0.047, *t* = −2.088, *p* = 0.038), supporting H4. Simple slope analyses were conducted at one standard deviation below and above the mean of LMX. When LMX was low (−1 SD), the association between telecommuting and workplace loneliness was positive and significant (effect = 0.199, SE = 0.067, 95% CI [0.068, 0.330]). When LMX was high (+1 SD), the association was substantially weaker and the confidence interval included zero (effect = 0.002, SE = 0.065, 95% CI [−0.126, 0.131]). This moderating pattern is illustrated in Figure 2.

**Table 6:** Moderation and moderated mediation results for leader–member exchange.

Panel A. Moderation analysis				
Predictor	*β*	SE	*t*	*p*
Telecommuting	0.100	0.046	2.169	0.031
Leader–member exchange	−0.374	0.059	−6.316	<0.001
Telecommuting × Leader–member exchange	−0.098	0.047	−2.088	0.038

Conditional effect of telecommuting on workplace loneliness				
Leader–member exchange level	Effect	SE	LLCI	ULCI
Low (−1 SD)	0.199	0.067	0.068	0.330
Mean	0.100	0.046	0.009	0.191
High (+1 SD)	0.002	0.065	−0.126	0.131

Panel B. Moderated mediation analysis				
Conditional indirect effect of telecommuting on cyberloafing through workplace loneliness				
Leader–member exchange level	Indirect effect	Boot SE	BootLLCI	BootULCI
Low (−1 SD)	0.075	0.026	0.029	0.132
Mean	0.038	0.019	0.007	0.082
High (+1 SD)	0.001	0.025	−0.045	0.054

Index of moderated mediation				
Moderator	Index	Boot SE	BootLLCI	BootULCI
Leader–member exchange	−0.037	0.017	−0.072	−0.005

Bootstrap sample size = 5,000; LLCI = lower limit confidence interval; ULCI = upper limit confidence interval; BootLLCI = bootstrap lower limit confidence interval; BootULCI = bootstrap upper limit confidence interval.

**Figure 2:**
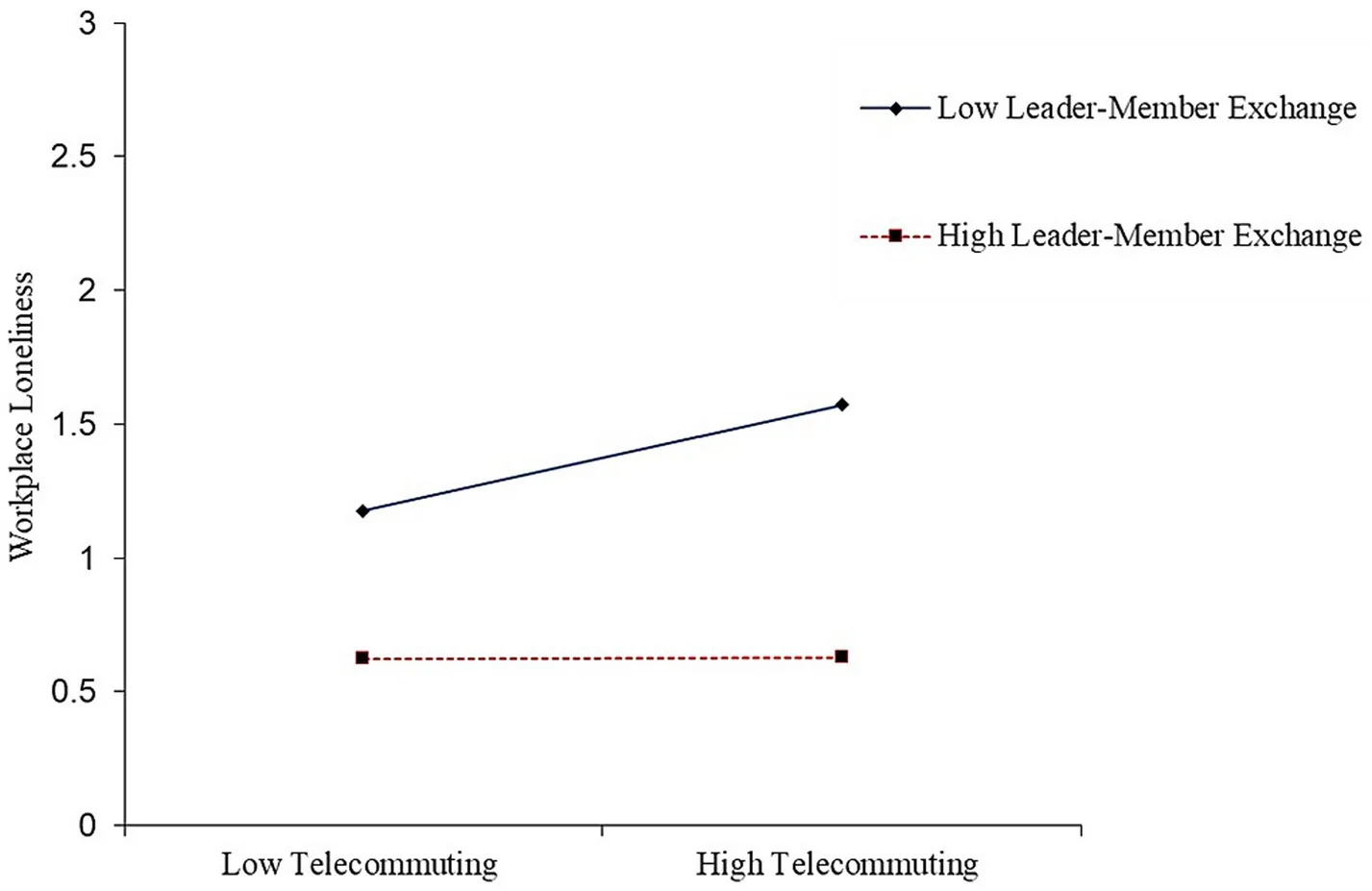
Moderating effect of leader–member exchange on the association between telecommuting and workplace loneliness. The figure illustrates the association between telecommuting and workplace loneliness at lower and higher levels of leader–member exchange.

The conditional indirect effect of telecommuting on cyberloafing through workplace loneliness also varied across levels of LMX. When LMX was low, the indirect effect was 0.075 (Boot SE = 0.026, 95% CI [0.029, 0.132]), whereas when LMX was high, the indirect effect was 0.001 (Boot SE = 0.025, 95% CI [−0.045, 0.054]). The index of moderated mediation was −0.037 (Boot SE = 0.017, 95% CI [−0.072, −0.005]). Because the 95% bootstrap confidence interval did not include zero, H5 was supported.

As shown in Table 7, the interaction between telecommuting and task interdependence was significant (*β* = −0.122, SE = 0.051, *t* = −2.373, *p* = 0.018), supporting H6. When task interdependence was low (−1 SD), the association between telecommuting and workplace loneliness was positive and significant (effect = 0.234, SE = 0.073, 95% CI [0.091, 0.377]). When task interdependence was high (+1 SD), the association was weaker and the confidence interval included zero (effect = −0.009, SE = 0.072, 95% CI [−0.150, 0.132]). This moderating pattern is illustrated in Figure 3.

**Table 7:** Moderation and moderated mediation results for task interdependence.

Panel A. Moderation analysis				
Predictor	*β*	SE	*t*	*p*
Telecommuting	0.112	0.051	2.219	0.027
Task interdependence	−0.215	0.057	−3.746	<0.001
Telecommuting × Task interdependence	−0.122	0.051	−2.373	0.018

Conditional effect of telecommuting on workplace loneliness				
Task interdependence level	Effect	SE	LLCI	ULCI
Low (−1 SD)	0.234	0.073	0.091	0.377
Mean	0.112	0.051	0.013	0.212
High (+1 SD)	−0.009	0.072	−0.150	0.132

Panel B. Moderated mediation analysis				
Conditional indirect effect of telecommuting on cyberloafing through workplace loneliness				
Task interdependence level	Indirect effect	Boot SE	BootLLCI	BootULCI
Low (−1 SD)	0.088	0.030	0.035	0.153
Mean	0.042	0.020	0.008	0.090
High (+1 SD)	−0.003	0.027	−0.056	0.052

Index of moderated mediation				
Moderator	Index	Boot SE	BootLLCI	BootULCI
Task interdependence	−0.046	0.020	−0.088	−0.009

Bootstrap sample size = 5,000; LLCI = lower limit confidence interval; ULCI = upper limit confidence interval; BootLLCI = bootstrap lower limit confidence interval; BootULCI = bootstrap upper limit confidence interval.

**Figure 3:**
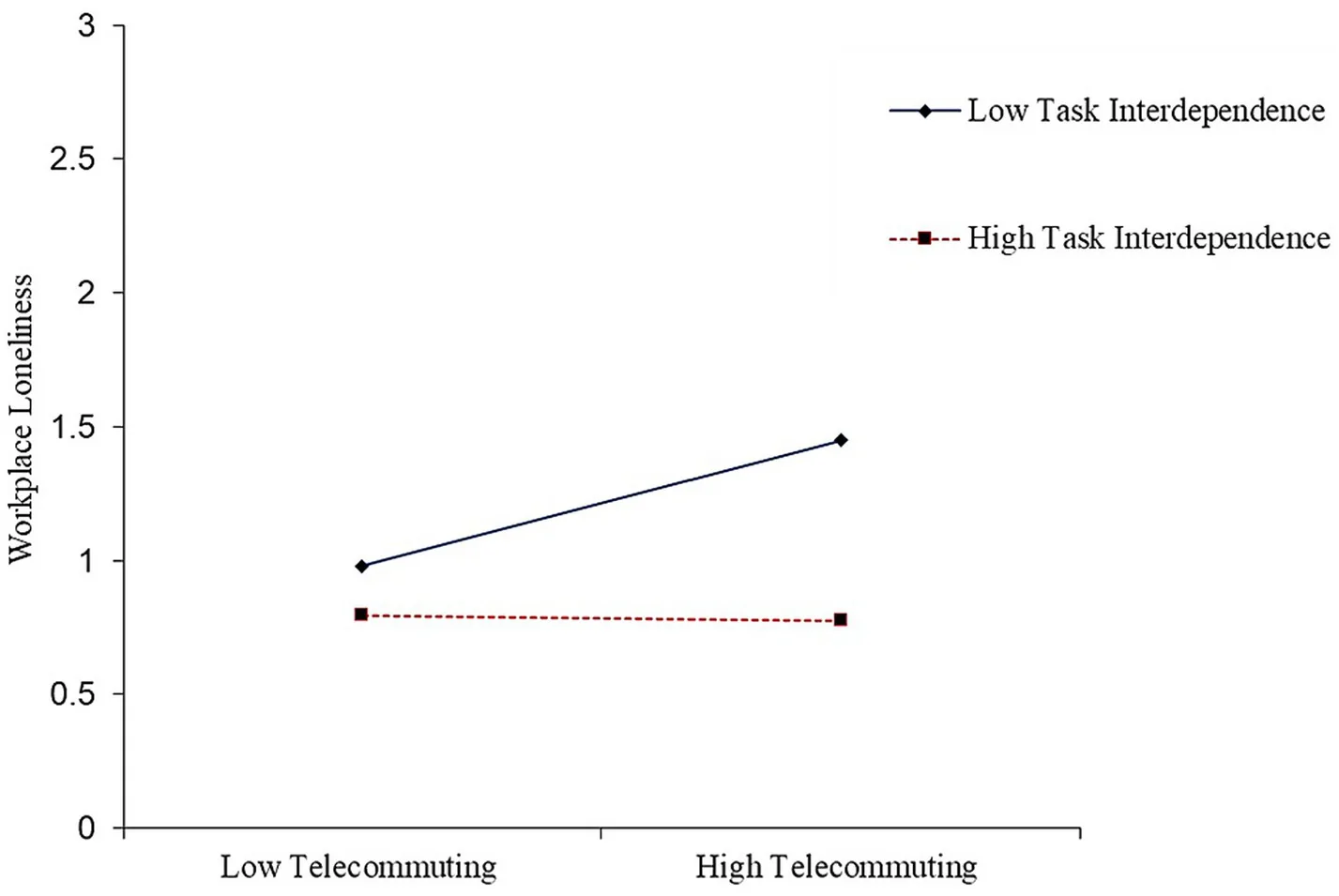
Moderating effect of task interdependence on the association between telecommuting and workplace loneliness. The figure illustrates the association between telecommuting and workplace loneliness at lower and higher levels of task interdependence.

The conditional indirect effect through workplace loneliness also varied across levels of task interdependence. When task interdependence was low, the indirect effect was 0.088 (Boot SE = 0.030, 95% CI [0.035, 0.153]), whereas when task interdependence was high, the indirect effect was −0.003 (Boot SE = 0.027, 95% CI [−0.056, 0.052]). The index of moderated mediation was −0.046 (Boot SE = 0.020, 95% CI [−0.088, −0.009]). Because the 95% bootstrap confidence interval did not include zero, H7 was supported.

## Discussion

Drawing on Conservation of Resources (COR) theory, this study examined how telecommuting is associated with cyberloafing through workplace loneliness and under what conditions this indirect association becomes weaker. The findings suggest that the implications of telecommuting cannot be understood simply in terms of physical separation from the workplace. Rather, what appears to matter is whether reduced physical co-presence is accompanied by diminished access to everyday social resources, a concern that has also been highlighted in research on telecommuting, professional isolation, and remote-worker wellbeing (Charalampous et al., 2019; Wang et al., 2020). In this process, workplace loneliness represents one psychological pathway through which diminished workplace connection may become associated with cyberloafing, rather than the sole mechanism underlying this relationship (Ozcelik and Barsade, 2018; McCarthy et al., 2026). This interpretation is also consistent with research showing that cyberloafing may occur in contexts of workplace strain, boredom, and resource depletion rather than solely as intentional misconduct (Andel et al., 2019; Pindek et al., 2018; Zhang et al., 2022).

At the same time, the present design does not allow the direction of the association between workplace loneliness and cyberloafing to be determined with confidence. A reciprocal relationship is also possible, as greater cyberloafing may be associated with weaker workplace connection and greater workplace loneliness. By linking telecommuting, workplace loneliness, and cyberloafing in this way, the study shifts attention from whether remote work is inherently beneficial or detrimental to how employees respond when valued social resources become less accessible.

The findings further show that this process is not uniform across employees or work contexts. Both LMX and task interdependence moderated the association between telecommuting and workplace loneliness, as well as the corresponding indirect association with cyberloafing. Although the two moderators showed broadly similar buffering patterns, the moderating effect associated with task interdependence was numerically somewhat larger than that associated with LMX. More importantly, they reflect different resource bases: LMX provides relational resources through supervisor support, trust, and socio-emotional attention (Varma et al., 2022), whereas task interdependence embeds interaction and coordination into the structure of work itself (Cheng et al., 2023; D’Oliveira and Persico, 2023). This distinction suggests that workplace connection under telecommuting can be sustained not only through the quality of interpersonal relationships, but also through the way work is organized.

However, neither LMX nor task interdependence should be viewed as an unconditional resource. High-quality LMX can provide support, trust, and access to relational resources, but it may also involve stronger expectations of reciprocity and additional obligations. Likewise, task interdependence can sustain workplace interaction while simultaneously increasing coordination and communication demands (Raveendran et al., 2020). Their resource value may therefore depend on whether the supportive and connective benefits they provide outweigh the demands they may also create. In the present context, the observed buffering patterns are consistent with a resource-enhancing role, while future research should examine the conditions under which these characteristics may instead function as demands.

The Chinese cultural context may also be relevant to the interpretation of these findings. In a context where collectivist and relational orientations, as well as hierarchical workplace relationships, may increase the salience of workplace social ties, reduced social connection may be experienced as particularly meaningful. High-quality LMX may therefore represent an especially salient relational resource, while task interdependence may help preserve connection through recurring work-related interaction. Because these cultural characteristics were not directly measured, however, they should be regarded as contextual considerations rather than tested explanations for the observed effects.

These findings complement prior research showing that flexible work arrangements can heighten workplace loneliness and reduce knowledge sharing under conditions of low task interdependence (Cheng et al., 2023). The present study addresses a related but distinct question through a resource-based account of telecommuting, showing that diminished workplace connection may also be associated with digital withdrawal and that both relationship-based and work-design-based resources can buffer this process. In this sense, the study offers a more differentiated account of remote work by showing that its social and behavioral consequences depend on the availability and form of resources that remain accessible when employees work apart.

### Theoretical implications

This study makes three theoretical contributions to research on telecommuting and employee digital behavior. First, rather than treating cyberloafing simply as another undesirable outcome of telecommuting, this study positions it as a possible form of short-term digital withdrawal under conditions of diminished workplace social connection. Telecommuting research has shown that employees’ experiences depend substantially on the relational conditions surrounding remote work (Golden, 2006), while more recent evidence continues to demonstrate that the consequences of telecommuting vary across work conditions and employee responses (Wang and Dong, 2025). The present findings add a different behavioral perspective: when working apart is accompanied by weakened workplace connection, non-work online activity may reflect temporary disengagement from the work environment rather than only deliberate rule violation. This interpretation is consistent with evidence linking cyberloafing to workplace strain and boredom (Andel et al., 2019; Pindek et al., 2018). The contribution therefore lies less in adding cyberloafing to the list of telecommuting outcomes than in extending attention to digital withdrawal as one possible behavioral response to diminished workplace social resources.

Second, the study specifies how diminished social resources under telecommuting may become psychologically consequential. Workplace loneliness is not treated merely as another adverse outcome of remote work, but as a subjective psychological experience of insufficient workplace connection that links reduced access to social resources with digital disengagement. Workplace relationships constitute an important source of everyday social and emotional resources (Methot et al., 2016), and research has shown that loneliness at work can shape both individual wellbeing and broader work functioning (Yang and Wen, 2021; Basit and Nauman, 2023). Building on COR theory, our findings suggest a more specific process: reduced access to informal interaction and relational contact may be associated with workplace loneliness, which is itself associated with a greater tendency toward digital withdrawal from work. Workplace loneliness thus represents one psychological pathway through which changes in the social conditions of telecommuting may become reflected in employees’ everyday digital behavior.

Third, the findings reveal that social connection under telecommuting can be preserved through two theoretically distinct resource pathways. High-quality LMX provides a relationship-based buffer, because trust, feedback, and socio-emotional support allow employees to remain connected to important workplace relationships even when physical access to supervisors is reduced (Sparr and Sonnentag, 2008). Task interdependence, by contrast, provides a work-design-based buffer, because communication and coordination are built into task accomplishment itself. Research on remote collaboration similarly shows that the organization of work can fundamentally shape interaction patterns when employees are geographically dispersed (Yang et al., 2022). This distinction helps explain why physical separation does not necessarily become social disconnection: employees may remain connected either because important relationships continue to provide social resources or because the structure of work continuously creates opportunities for interaction. Together, LMX and task interdependence identify relationship-based and work-design-based pathways through which access to workplace social resources may be maintained under telecommuting. This resource-buffering account offers a more differentiated explanation of why the social consequences of telecommuting vary across employees and work settings.

### Practical implications

The findings suggest that organizations should address cyberloafing by strengthening the social and relational conditions that help employees remain connected while telecommuting, rather than relying primarily on monitoring, restrictions, or surveillance-oriented responses (Lim and Teo, 2005). Given the modest but significant indirect association through workplace loneliness, remote-work practices may benefit from preserving regular opportunities for employees to remain connected with coworkers and supervisors. For example, teams can establish predictable check-ins, brief informal exchanges, and recurring opportunities for employees to discuss both task progress and work-related concerns. Such practices should support meaningful connection without creating unnecessary communication demands, particularly because effective remote work depends not only on technological access, but also on maintaining communication and social support (Wang et al., 2021).

The moderating patterns associated with LMX and task interdependence further point to two practical avenues: relational support and work-design support. Supervisors should maintain responsive one-to-one communication, provide timely feedback, and remain accessible when remote employees need support, rather than relying only on formal task supervision (Varma et al., 2022). At the same time, organizations should design remote workflows so that jobs requiring collaboration include clear coordination points, shared tasks, and regular information exchange. For relatively independent roles, organizations should not artificially increase task interdependence merely to create social contact; instead, they can provide alternative opportunities for meaningful work-related interaction. Together, these practices can help preserve relational and work-design resources while avoiding unnecessary monitoring or communication burdens.

### Limitations and future research

Several limitations should be acknowledged. First, although the two-wave time-lagged design introduced temporal separation between the predictor and outcome-related variables, workplace loneliness and cyberloafing were measured at the same wave. Thus, the temporal ordering between the mediator and outcome cannot be fully established, and reverse or reciprocal relationships between workplace loneliness and cyberloafing cannot be ruled out. In addition, the two-week interval was designed to capture relatively proximal associations and may not reflect longer-term adaptation to telecommuting. Future research could employ three-wave longitudinal or experimental designs with varying or longer time intervals to provide stronger evidence for the proposed process and examine how these relationships develop over time.

Second, the study relied on a convenience sample of employees in China, which may limit the generalizability of the findings to other national and cultural contexts. Cultural differences in collectivism, workplace hierarchy, and relational orientation may influence the salience of workplace loneliness, LMX, and task interdependence. Future research should therefore examine whether the observed relationships replicate across different cultural and institutional settings.

Finally, telecommuting was assessed using a single-item measure of weekly remote-work hours, which captures telecommuting intensity but may not fully represent other dimensions of remote-work arrangements or employees’ broader experiences of telecommuting. Future research could examine whether the findings generalize across more comprehensive measures of telecommuting. Relatedly, employees’ experiences of telecommuting may also depend on how workplace interaction is maintained through digital communication. Future research could therefore examine whether communication richness, the quality and frequency of virtual interaction, or digital overload shape the relationships among telecommuting, workplace loneliness, and cyberloafing. In addition, cyberloafing was assessed using self-reported data and may therefore be subject to social desirability bias and potential underreporting. Future studies could complement self-reports with behavioral indicators, digital trace data, or multi-source evaluations to further validate cyberloafing behaviors.

## Data Availability

The raw data supporting the conclusions of this article will be made available by the authors, without undue reservation.
